# Early microvascular changes in the preterm neonate: a comparative study of the human and guinea pig

**DOI:** 10.14814/phy2.12145

**Published:** 2014-09-17

**Authors:** Rebecca M. Dyson, Hannah K. Palliser, Anil Lakkundi, Koert de Waal, Joanna L. Latter, Vicki L. Clifton, Ian M. R. Wright

**Affiliations:** 1 Mothers and Babies Research Centre, Hunter Medical Research Institute, Newcastle, NSW, Australia; 2 Discipline of Paediatrics and Child Health, School of Medicine and Public Health, University of Newcastle, Newcastle, NSW, Australia; 3 Graduate School of Medicine and Illawarra Health and Medical Research Institute, University of Wollongong, Wollongong, NSW, Australia; 4 School of Biomedical Sciences and Pharmacy, University of Newcastle, Newcastle, NSW, Australia; 5 Kaleidoscope Neonatal Intensive Care Unit, John Hunter Children's Hospital, Newcastle, NSW, Australia; 6 Robinson Institute, School of Paediatrics and Reproductive Health, University of Adelaide, Adelaide, SA, Australia

**Keywords:** Guinea pig, hypoperfusion–reperfusion, microvascular blood flow, preterm neonate

## Abstract

Dysfunction of the transition from fetal to neonatal circulatory systems may be a major contributor to poor outcome following preterm birth. Evidence exists in the human for both a period of low flow between 5 and 11 h and a later period of increased flow, suggesting a hypoperfusion–reperfusion cycle over the first 24 h following birth. Little is known about the regulation of peripheral blood flow during this time. The aim of this study was to conduct a comparative study between the human and guinea pig to characterize peripheral microvascular behavior during circulatory transition. Very preterm (≤28 weeks GA), preterm (29–36 weeks GA), and term (≥37 weeks GA) human neonates underwent laser Doppler analysis of skin microvascular blood flow at 6 and 24 h from birth. Guinea pig neonates were delivered prematurely (62 day GA) or at term (68–71 day GA) and laser Doppler analysis of skin microvascular blood flow was assessed every 2 h from birth. In human preterm neonates, there is a period of high microvascular flow at 24 h after birth. No period of low flow was observed at 6 h. In preterm animals, microvascular flow increased after birth, reaching a peak at 10 h postnatal age. Blood flow then steadily decreased, returning to delivery levels by 24 h. Preterm birth was associated with higher baseline microvascular flow throughout the study period in both human and guinea pig neonates. The findings do not support a hypoperfusion–reperfusion cycle in the microcirculation during circulatory transition. The guinea pig model of preterm birth will allow further investigation of the mechanisms underlying microvascular function and dysfunction during the initial extrauterine period.

## Abstract (precis)

e12145

A period of high microvascular flow occurs after birth, however, no period of low flow (hypoperfusion stage) precedes this, suggesting that no hypoperfusion–reperfusion cycle exists during early extrauterine life. The data strongly support the use of the preterm guinea pig in future studies to allow further investigation of the interrelationship between cardiac output, preload, afterload, microvascular flow, and SVC flow. Elucidation of these processes may then aid clinicians in managing the circulatory dysfunction of preterm neonates, particularly those at greatest risk, preterm male neonates.

## Introduction

Many complications of prematurity, including neurodevelopmental deficits, have been hypothesized to have ischemic origins (Evans ^10^). These complications may be a result of the preterm neonate's failure to effectively transition from a fetal to a neonatal circulatory system (Sinha and Donn ^30^). A significant relationship between microvascular dilatation, mean arterial pressure, and poor outcome has previously been demonstrated in a preterm neonatal population. Abnormal microvascular tone, characterized by inappropriate vasodilatation and thus high blood flow throughout peripheral tissues, may contribute to the development of circulatory compromise in the preterm neonate (Stark et al. ^32^; Ishiguro et al. ^15^; Schwepcke et al. ^29^). High microvascular blood flow at 24 h is associated with cardiorespiratory instability and adverse outcome in the first 72 h of postnatal life (Stark et al. ^32^).

Male sex is an independent risk factor for poor outcome following preterm birth: male neonates are at much greater risk of dying or suffering from neurodevelopmental disability (Ambalavanan et al. ^2^). In our nursery, the male death rate at <29 weeks gestational age (GA) is more than double that of females (26% vs. 12%). Male morbidity is shown by a 13% increased length of stay and increased readmission in the first year of life (Kent et al. ^17^; Abdel‐Latif et al. ^1^). Sexual dimorphism in the functional integrity of the microvasculature, including appropriate control of vasodilatation both at baseline and in response to stimuli, has been identified in very preterm neonates. Significantly higher levels of baseline microvascular blood flow are observed at 24 h postnatal age in males compared to females of the same gestational age and compared to males born at later gestational ages (Stark et al. ^33^), suggesting a gestational age‐dependent, sex‐specific difference in the neonatal ability to control vascular tone.

These changes have been observed in discreet clinical studies with defined time points. Ongoing assessment of microvascular blood flow over the first few days of postnatal life – the period of circulatory transition and significant change in the neonatal circulatory system – in both preterm and term neonates has not been conducted. Recent studies have examined microvascular blood flow changes in very low birth weight neonates during this period (Ishiguro et al. ^15^). These studies, consistent with earlier reports, found that skin microvascular blood flow increased from birth. However, changes in microvascular blood flow (both baseline and in response to stimuli) were different between the forehead and the lower limb, with lower limb blood flow apparently a more sensitive measure of circulatory changes during transition (Ishiguro et al. ^16^).

Measurement of superior vena caval (SVC) flow in preterm neonates (Evans et al. ^12^) identified low blood flow within the first 12 h of postnatal life in approximately one‐third of neonates delivered prior to 30 weeks completed gestation (Evans ^10^). This period of postnatal hypoperfusion is followed by an increase in SVC flow at 24 h and associated with adverse clinical outcome. Similarly, the peripheral microvasculature also has increased flow at 24 h postnatal age and is associated with adverse clinical outcome (Stark et al. ^32^,b). Although systemic macrovascular flow changes have been assessed, previous studies have not investigated early microvascular flow changes.

These macrovascular observations have led to the suggestion of a hypoperfusion–reperfusion cycle over the first day of postnatal life, where, in the first 12 h there is a state of overall vasoconstriction and low flow, followed by a state of uncontrolled vasodilatation and high flow at 24 h, which stabilizes by 72 h postnatal age (Evans ^10^; Stark et al. ^32^). As such, the aim of this study was to investigate microvascular blood flow at 6, 24, and 72 h postnatal age in the human neonate. It was hypothesized that early microvascular flow (6 h) would be low, with a significant increase to 24 h and that this effect would be most obvious in the highest risk groups, that is, very preterm males.

Noninvasive multiple measurements are not suitable in the very preterm human infants so we also sought to compare our findings in an established preterm guinea pig animal model (Dyson et al. ^7^). The guinea pig model was used to characterize microvascular blood flow changes occurring over the first day of postnatal life. It was hypothesized that a similar cycle of hypoperfusion–reperfusion would be observed in the preterm neonatal guinea pig as in the human neonate. Further characterization of early changes in microvascular function in this animal model will allow for subsequent investigations of mechanisms underpinning cardiovascular maladaptation in the preterm neonate.

## Materials and Methods

### Human neonates

#### Subjects

Expectant mothers were recruited into the “Cardiovascular Adaptation of the Newborn Study 2 (2CANS)” after informed consent following presentation to the John Hunter Hospital, Newcastle, Australia with threatened premature delivery. Women for term controls were recruited from the antenatal clinic during their pregnancy and included if their pregnancy proceeded to term. The study was stratified by gestational age at delivery (very preterm: ≤28 weeks, preterm 29–36 weeks, term ≥37 weeks), based on our previous study (CANS1) (Stark et al. ^32^,b). Hypoxic ischemic encephalopathy, congenital malformations, chromosomal disorders, or known congenital infection excluded admission to this study. All study protocols were approved by the local Ethics Committees at the John Hunter Hospital (Hunter New England Area Health Service) and University of Newcastle.

#### Microvascular studies

Peripheral microvascular blood flow in the skin was assessed at 6, 24, and 72 h postnatal age by laser Doppler flowmetry (LDF) using a Periflux 5001 Laser Doppler (Perimed AB, Jarfalla, Sweden) as in our previous studies (Stark et al. ^32^,b). Briefly, temperature‐regulated Doppler probes (Probe 457, Perimed AB) were positioned on the lower limb surface. Basal blood flow was recorded for 5 min, followed by a standard occlusive challenge, enabling comparison between different time points and subjects.

#### Cardiovascular studies

Cardiovascular studies were performed for preterm infants only. Mean arterial blood pressure, measured by in‐dwelling arterial catheter or noninvasive oscillometric recording, was determined from averaged serial blood pressure readings over a 30 min period immediately prior to laser Doppler studies. Previous studies have established an adequate level of agreement between these two methodologies in this population (Roeder and Geddes ^26^).

Echocardiographic studies were performed by two investigators (KW and AL) immediately after microvascular studies using an iE33 ultrasound system (Philips Healthcare, DA Best, The Netherlands) with a 12 MHz vector array transducer. All Doppler measurements were performed according to previously published methodology (Evans and Kluckow ^11^; Kluckow and Evans ^18^). Low blood flow was defined as a SVC flow <45 mL/kg/min or a right ventricular output (RVO) or left ventricular output (LVO) <150 mL/kg/min.

Blood gas analysis was performed for analysis of blood pH, base excess and lactate using an ABL700 blood gas analyzer (Radiometer, Copenhagen, Denmark). Clinical illness severity was assessed with the Clinical Risk Index for Babies (CRIB) II scoring system (Parry et al. ^25^).

### Guinea pig neonates

#### Animals

The Research Support Unit of the University of Newcastle supplied time‐mated, pregnant outbred, tricolor guinea pigs. All procedures were approved by the University of Newcastle Animal Care and Ethics Committee and carried out in accordance with the Australian Code of Practice for the Care and Use of Animals for Scientific Purposes (Australian Government National Health and Medical Research Council ^3^). Male and female offspring were used for this study, aiming for no more than one pup of each sex from each dam.

#### Delivery & neonatal support

Dams were allocated to spontaneous term delivery, caesarean section term delivery or caesarean section preterm delivery. All pregnant dams received betamethasone (1 mg/kg Celestone Chronodose, Schering‐Plough Corporation, Kenilworth, NJ) at 24 and 12 h prior to delivery for dams undergoing caesarean section to mimic the human preterm delivery protocol (Dyson et al. ^7^).

Preterm animals were delivered by caesarean section at 62 ± 1 days GA. We have previously determined that neonates delivered at this gestational age are functionally equivalent to a human neonate of approximately 29 weeks gestation and display similar changes in respiratory and microvascular behavior to the very preterm human (Dyson et al. ^7^). This gestational age equivalency is supported by earlier studies that demonstrated that guinea pig pups born at 0.88 gestation had lung morphology indicative of the saccular stage of lung development (observed in humans from approximately 24 weeks gestational age) and that these pups cannot survive without significant intensive care interventions (Engel ^9^; Sosenko and Frank ^31^). Guinea pig neonates cannot survive before GA61 without invasion interventions (intubation), thus GA62 was selected to reflect our clinical studies (no intubated neonates were included in the human studies).

Term controls were delivered by caesarean section at GA69 or a day earlier if the pubic symphysis had been fully open for two consecutive days. Delivery was performed as previously described (Dyson et al. ^7^). Briefly, the uterus was exposed and removed from the abdominal cavity maintaining blood supply. Pups were rapidly removed from the uterus to a warm bed for immediate resuscitation. Following delivery, preterm pups received surfactant (Curosurf, 80 mg/mL Poractant Alfa, Ascent Pharmaceuticals Ltd, Victoria, Australia) and respiratory support (suction, stimulation, CPAP, IPPV by mask and increasing FiO_2_ as required. Endotracheal ventilation was not undertaken). Once stable, term and preterm pups were maintained in a humidified incubator at temperatures of 34–38°C as required for maintenance of body temperature for the duration of the study period. Oral temperature was measured two‐hourly at time of laser Doppler measurement. Neonatal well‐being was scored using a model‐specific neonatal monitoring score, with higher scores reflecting greater physiological stability (Dyson et al. ^7^). Pups were fed two hourly, immediately following laser Doppler assessment.

Dams allocated to the spontaneous delivery group were monitored weekly until the identification of full pubic symphysis separation, indicating early labor. At this time dams were administered betamethasone, with a second dose administered 12 h later if labor had not occurred and monitored continuously by infrared camera. Pups were immediately separated from dams following delivery and monitored, housed and fed as detailed above for pups delivered by caesarean section.

#### Microvascular studies

Laser Doppler assessment of microvascular blood flow was performed two hourly from birth until postmortem between 22 and 24 h postnatal age using the Periflux System 5001 (Perimed) with small thermostatic probe (Probe 457, Perimed) attached. At birth, a 20 mm × 20 mm area at the nape of the neck was shaved and an electrode ring (tcpO2/tcpCO2 fixation ring; Philips, Boblingen, Germany) was affixed to the skin using veterinary grade tissue adhesive (Vetbond; 3M, St Paul, MN) to ensure serial measurement of microvascular blood flow was kept at a constant location over the entirety of the study.

At the time of recording, the probe was fixed into the probe holder located at the nape of the neck. The probe was heated to 36°C and baseline blood flow was recorded for a minimum of 1 min. Pinching the skin at the back of the neck and occluding blood flow to the subcutaneous microcirculation below the probe for 1 min allowed a biological zero to be obtained for each recording. As in the human studies, this zero was subtracted from the baseline blood flow in each recording to give the reported value. Peripheral microvascular blood flow recordings were analyzed offline using custom software (Perisoft 1.14; Perimed AB). Only recording segments free from movement artifacts were analyzed.

### Statistics

Stata 13 for MacOSX (StataCorp LP, College Station, TX) was used for statistical analyses. Stata 13 and Prism 6 for MacOSX GraphPad Software Inc., La Jolla, CA) were used for generation of graphs. Unless otherwise stated, data for physical characteristics are presented as median (range) and analyzed via Mann–Whitney Rank Sum test. Incidence and mortality data were analyzed using Fisher's Exact Test. Spearman r tests were used to investigate correlations between blood flow, physiological stability, and cardiorespiratory parameters. As in previous studies, LDF data were not normally distributed and were transformed using natural logarithm for analysis of serial microvascular blood flow measurement (log_e_PU). Linear mixed regression was used to investigate the association of blood flow with gestational and postnatal age, sex, and mode of delivery in guinea pig neonates. Blood flow changes over time were analyzed via random effects generalized least squares regression with bootstrapping (1000 repetitions). The level of statistical significance for all analyses was set at *P *≤ 0.05 using two‐tailed comparisons.

## Results

### Human neonates

One hundred and thirty‐eight neonates (46 very preterm, 50 preterm, and 42 term) were studied and their clinical characteristics are shown in Table 1. Based on a priori stratification, there were significant differences between gestational age groups for birth weight (males *P* = 0.0001, females *P* = 0.0001), exposure to antenatal corticosteroids (males: *P* = 0.0001, females *P* = 0.0003), APGAR score at 5 min (males *P* = 0.003, females *P* = 0.0001), CRIB II score (males *P* = 0.0001, females *P* = 0.0001), PDA (males *P* = 0.02, females *P* = 0.01), sepsis (males only, *P* = 0.0009), and mortality (males *P* = 0.004, females *P* = 0.02). Despite stratification, the females in the very preterm group were younger and lighter than the males (gestational age, *P* = 0.009; birth weight, *P* = 0.02; see Table 1).

**Table 1.** Clinical characteristics of human neonates.

	Very preterm group		Preterm group		Term group	
	Female (*n*=23)	Male (*n*=23)	Female (*n*=23)	Male (*n*=27)	Female (*n*=20)	Male (*n*=22)
Gestation (week)	26 (24–28)	27 (24–28)*	32 (29–36)	31 (29–35)	39 (38–41)	39 (38–43)
Birth weight (kg)	0.83 (0.45–1.38)	1.01 (0.56–1.40)*	1.76 (0.97–3.89)	1.56 (0.58–2.76)	3.37 (2.66–4.12)	3.68 (2.53–4.32)
Small for gestational age (*n*, %)	1 (4%)	3 (13%)	0	4 (15%)	2 (10%)	2 (9%)
Completed antenatal steroids (*n*, %)^1^	16 (70%)	17 (74%)	14 (61%)	20 (74%)	2 (10%)	1 (4.5%)
5‐min APGAR score	8 (4–10)	8 (4–10)	9 (5–10)	9 (5–10)	9 (9–10)	9 (9–10)
CRIB II score	12 (8–15)	10 (7–16)	3 (1–8)	4 (1–11)	0 (0–3)^2^	0
Patent ductus arteriosus (*n*, %)	11 (48%)	7 (30%)	1 (4%)	5 (19%)	0	0
Sepsis (*n*, %)	9 (39%)	10 (43%)	3 (13%)	4 (15%)	0	0
Necrotizing enterocolitis (*n*, %)	3 (13%)	1 (4%)	0	0	0	0
IVH >grade II (*n*, %)	1 (4%)	2 (9%)	0	0	0	0
Death (*n*, %)^3^	4 (17%)	5 (22%)	0	0	0	0

Data are presented as median (range) or number (%). APGAR Score – scores 7 and above are generally regarded as normal, 4–6 fairly low, and 3 and below critically low; CRIB II Score – Clinical Risk Index for Babies II, higher scores reflect poorer physiological stability; Patent Ductus Arteriosus refers to a hemodynamically significant duct diagnosed in first 72 h; IVH – intraventricular hemorrhage greater than grade II (significant IVH).

* Significant difference from females of the same gestational age group, *P* < 0.05.

^1^ Completed antenatal steroids refers to neonates who received a full course of antenatal betamethasone (two injections, 24 h apart) only and does not include neonates born to mothers who only received one dose prior to delivery.

^2^ Only one term female was evaluated for CRIB score. If CRIB assessment not completed, well‐term infants were assigned a value of 0.

^3^ Death rate does not include stillborns and refers to neonatal deaths only – that is, live born neonates who did not survive.

Pregnancy and maternal characteristics are presented in Table 2. There was a higher rate of multiple births (twins and triplets) in the preterm groups (*P* < 0.0001) compared to term (no multiple births). There was also a higher rate of multiple births of males in the very preterm group (*P* = 0.01 compared to females of the same gestational age group).

**Table 2.** Maternal characteristics and pregnancy outcomes.

	Very preterm group		Preterm group		Term group	
	Female (*n*=23)	Male (*n*=23)	Female (*n*=23)	Male (*n*=27)	Female (*n*=20)	Male (*n*=22)
Maternal BMI	25 (19–43)	25 (19–44)	27 (19–42)	22 (18–41)	24 (19–39)	26 (17–40)
Maternal smoking (*n*, %)	6 (26%)	5 (22%)	5 (22%)	5 (19%)	0	3 (13%)
Pregnancy‐induced hypertension (*n*, %)	3 (13%)	4 (17%)	10 (43%)	7 (26%)	5 (25%)	4 (18%)
Gestational diabetes (*n*, %)	2 (9%)‡	0	1 (4%)‡	4 (15%)	1 (5%)‡	0
Vaginal delivery (*n*, %)	11 (48%)	9 (39%)	11 (48%)	14 (52%)	11 (55%)	12 (55%)
Singleton births (*n*, %)	20 (87%)	12 (52%)*	16 (70%)	14 (52%)	20 (100%)†	22 (100%)†
PROM (*n*, %)	8 (35%)	3 (13%)	6 (26%)	7 (26%)	1 (5%)	2 (9%)
Chorioamnionitis (*n*, %)	2 (9%)	1 (4%)	1 (4%)	2 (7%)	0	0

Data are presented as median (range) or number (%). PROM, prolonged rupture of membranes. Vaginal delivery reflects both assisted and unassisted deliveries.

* Significant difference from females of the same gestational age group, *P *= 0.01.

‡ Significant difference between gestational age groups, *P *< 0.05 within sex.

† Significant difference from preterm neonates (all <37 weeks), *P *< 0.0001.

#### Human microvascular studies

**Figure 1.**
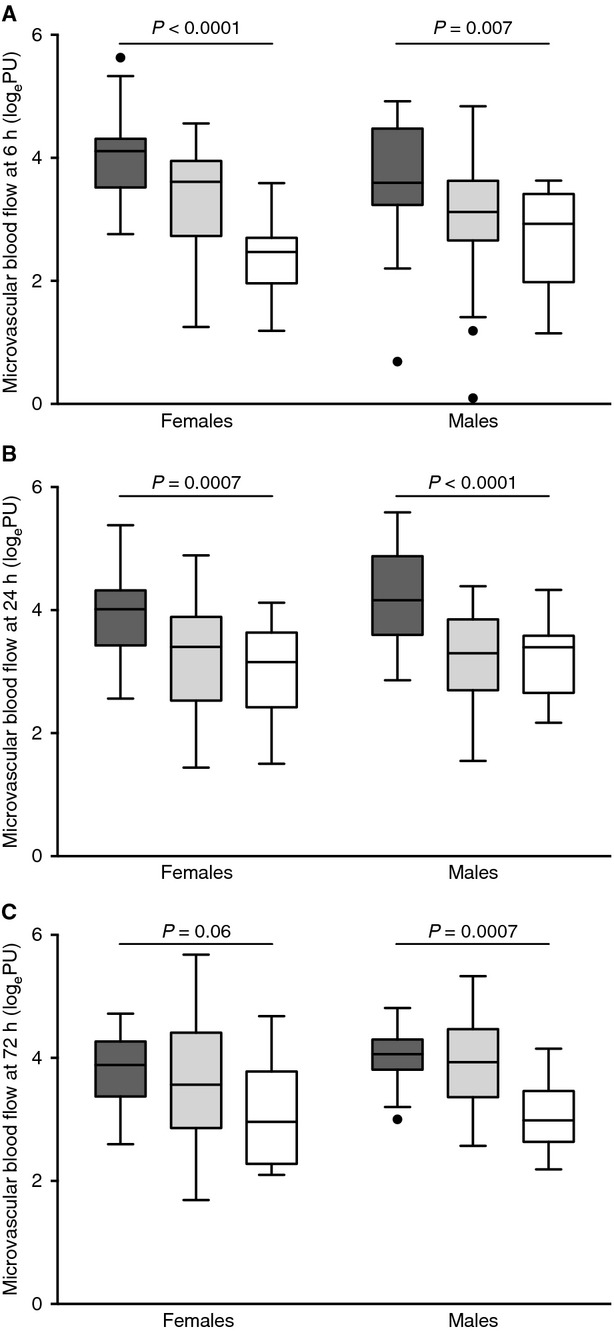
Microvascular blood flow as measured by laser Doppler flowmetry in very preterm (≤28 weeks gestational age; dark gray bars), preterm (29–36 weeks gestational age; light gray bars) and term (≥37 weeks gestational age; open bars) infants. (A) 6 h postnatal age. Microvascular blood flow was highest in very preterm neonates and decreased with advancing gestational age (females *P *< 0.0001, males *P *= 0.007). (B) 24 h postnatal age. As at 6 h, blood flow was highest in the most premature infants (females *P *= 0.0007, males *P *< 0.0001). (C) 72 h postnatal age. Gestational age differences in microvascular blood flow were observed only in males (*P *= 0.0007, females *P* = 0.06). Data are presented as Tukey box and whisker plots (median ± interquartile range [IQR] plus 1.5IQR. Values plotted individually fall outside this range).

At all time points studied, gestational age (weeks) was inversely correlated with microvascular blood flow (6 h females *P* < 0.0001, *r* = −0.68; 6 h males *P* = 0.0001, *r* = −0.46; 24 h females *P* < 0.0001, *r* = −0.53; 24 h males *P* < 0.0001, *r* = −0.48; 72 h females *P* = 0.008, *r* = −0.37; 72 h males *P* = 0.0006, *r* = −0.45; Fig. 1).

**Figure 2.**
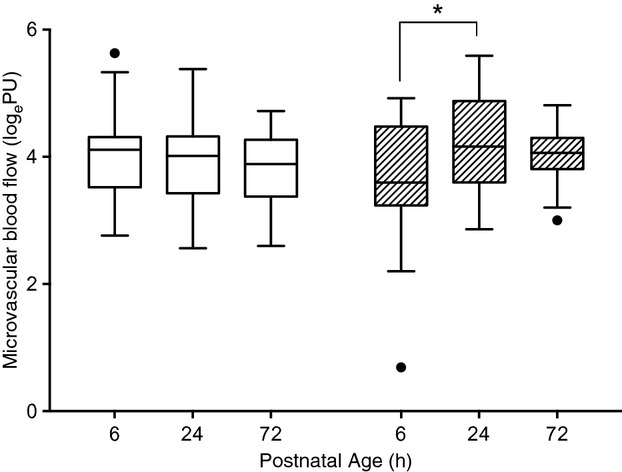
Microvascular blood flow changes over time in very preterm neonates. Microvascular blood flow increased from 6 to 24 h postnatal age in males (hatched bars) and then remained constant to 72 h (*P *= 0.19). No changes were observed in females (open bars; *P *= 0.69). *Significance, *P *= 0.01. Data are presented as Tukey box and whisker plots (median ± interquartile range [IQR] plus 1.5IQR. Values plotted individually fall outside this range).

In very preterm male neonates, microvascular flow increased from 6 to 24 h postnatal age (*P* = 0.01). There was no change from 24 to 72 h in this group (*P* = 0.19) nor in any other gestational age group. In females, there was no change in flow over time (*P* = 0.69; Fig. 2) at any gestational age studied.

Microvascular blood flow was significantly higher in preterm neonates (all neonates <37 weeks gestational age) born to mothers with chorioamnionitis (no chorioamnionitis 36.68 (4.22–266.8) PU; chorioamnionitis 79.16 (46.33–168.1) PU; *P* = 0.02). There was no effect of any other maternal or pregnancy‐related characteristics on microvascular blood flow (data not shown).

#### Human macrovascular studies

There was no evidence of a hypoperfusion stage in the preterm neonates as SVC flow at 6 h postnatal age was not significantly lower than at 24 h postnatal age; *P* = 0.22; and was not significantly lower in very preterm (<28 weeks) than preterm newborns (29–36 weeks) *P* = 0.18. In fact, the incidence of low SVC flow (defined as flow <45 mL/kg/min) was higher in preterm than very preterm newborns at 6 h postnatal age (*n* = 8 preterm neonates, *n* = 4 very preterm neonates; *P* = 0.05). As our microvascular blood flow data may represent an inappropriate distribution of blood flow through the periphery away from central organs, we examined the relationship between skin microvascular flow and SVC flow and found no relationship between the two at any time point for any group studied (data not shown). There was, however, a positive correlation between LVO and skin microvascular blood flow in neonates <37 weeks GA at 6 h (*P* = 0.026, *r* = 0.29) and 24 h (*P* = 0.07, *r* = 0.24). LVO was higher in very preterm males (≤28 weeks gestational age) than preterm males (29–36 weeks gestational age) at all times points studied, although this did not reach statistical significance until 72 h postnatal age (6 h *P* = 0.088; 24 h *P* = 0.083; 72 h *P* = 0.023; see Table 3).

**Table 3.** Cardiorespiratory parameters of preterm neonates over the first 3 days of life.

	Very preterm		Preterm	
	Females (*n*=23)	Males (*n*=23)	Females (*n*=23)	Males (*n*=27)
Total days O_2_	24 (0 to 493)‡	7 (0 to 356)‡	0 (0 to 350)	0 (0 to 576)
Total days IMV	0 (0 to 6)‡	0 (0 to 25)‡	0	0 (0 to 21)
Total days CPAP	34 (1 to 57)‡	36 (1 to 64)‡	1 (0 to 32)	1 (0 to 32)
Respiratory rate (breaths per minute)				
Admission	46 (24 to 76)‡	48 (30 to 72)	57 (38 to 80)	60 (32 to 100)
6 h	54 (36 to 38)	51 (39 to 76)	57 (38 to 70)	56 (46 to 96)
24 h	56 (40 to 85)	55 (42 to 74)	56 (43 to 66)	52 (40 to 64)
72 h	56 (43 to 64)	62 (44 to 70)*^,^*	52 (37 to 66)	52 (42 to 66)
Heart rate (bpm)				
Admission	160 (128 to 190)	156 (110 to 190)	150 (122 to 180)	149 (122 to 188)
6 h	144 (126 to 158)	141 (122 to 172)‡	140 (122 to 170)	130 (108 to 160)*
24 h	149 (126 to 162)‡	148 (123 to 179)‡	135 (118 to 168)	134 (112 to 164)
72 h	150 (136 to 166)	148 (130 to 194)	150 (115 to 172)	142 (118 to 160)
Mean blood pressure (mmHg)				
Admission	31 (26 to 45)	35 (32 to 37)	41 (40 to 41)	40 (28 to 53)
6 h	32 (26 to 43)‡	34 (25 to 48)‡	41 (27 to 53)	41 (29 to 55)
24 h	36 (24 to 44)‡	36 (26 to 51)	52 (34 to 68)	39 (30 to 81)*
72 h	36 (30 to 50)‡	40 (27 to 51)‡	52 (33 to 67)	49 (34 to 70)
SVC flow (mL/kg/min)				
6 h	73 (44 to 157)	74 (28 to 122)	73 (44 to 111)	65 (29 to 210)
24 h	81 (52 to 174)	86 (49 to 139)	87 (40 to 152)	100.5 (45 to 167)
72 h	100 (62 to 160)	88.5 (51 to 203)	84 (36 to 243)	73 (40 to 137)
RVO (mL/kg/min)				
6 h	255 (150 to 356)	237 (97 to 568)	217.5 (159 to 319)	222 (94 to 467)
24 h	278 (203 to 388)	282.5 (137 to 544)	267.5 (153 to 425)	256.5 (169 to 358)
72 h	316 (195 to 404)	348.5 (184 to 570)	275 (218 to 370)	267 (131 to 566)
LVO (mL/kg/min)				
6 h	226 (138 to 346)	178 (116 to 352)	209.5 (131 to 307)	150 (110 to 275)*
24 h	292 (169 to 433)	292 (145 to 409)	221 (106 to 298)	199.5 (96 to 324)
72 h	279 (170 to 483)	274.5 (160 to 478)‡	234 (137 to 321)	191 (111 to 415)
Blood pH				
6 h	7.34 (7.19 to 7.41)	7.33 (7.18 to 7.43)	7.32 (7.23 to 7.40)	7.35 (7.15 to 7.52)
24 h	7.37 (7.31 to 7.47)	7.34 (7.20 to 7.49)	7.35 (7.29 to 7.45)	7.35 (7.24 to 7.42)
72 h	7.30 (7.20 to 7.41)	7.29 (7.06 to 7.38)‡	7.32 (7.26 to 7.37)	7.32 (7.19 to 7.41)
Base excess (mEq/L)				
Admission	−5.1 (−13.2 to −1.0)	−6.4 (−13.8 to −0.9)‡	−3.6 (−6.3 to 2.3)	−2.2 (−15.6 to 4.3)
6 h	−3.7 (−9.9 to 0.8)	−3.4 (−9.9 to −0.7)‡	−3.2 (−5.0 to 2.1)	−1.2 (−6.3 to 4.4)
24 h	−3.9 (−7.2 to 3.3)	−4.2 (−11.0 to 5.2)‡	−3.1 (−7.0 to 2.9)	−1.7 (−6.0 to 4.3)
72 h	−8.5 (−12.2 to −3.1)‡	−8.0 (−17.2 to −4.8)‡	−4.4 (−7.5 to −2.5)	−4.0 (−7.3 to 0.3)
Blood lactate (mg/dL)				
6 h	1.9 (1.0 to 6.7)	1.9 (1.1 to 10.1)	2.2 (1.6 to 4.5)	2.2 (0.9 to 6.9)
24 h	1.7 (1.1 to 3.9)	2.2 (1.3 to 7.1)*	1.9 (1.4 to 4.4)	2.0 (1.3 to 5.6)
72 h	1.3 (0.9 to 2.0)	1.5 (0.7 to 11.1)	1.5 (0.9 to 2.2)	1.6 (0.9 to 2.3)

Data are presented as median (range). IMV, intermittent mandatory ventilation; CPAP, continuous positive air pressure respiratory support; SVC flow, superior vena caval blood flow; RVO, right ventricular output; LVO, left ventricular output.

* Significant difference from females of the same gestational age group, *P *< 0.05.

‡ Significant difference between gestational age groups, *P *< 0.05 within sex.

#### Human microvascular blood flow and physiological instability

Cardiorespiratory parameters for preterm neonates (<37 weeks gestation) are shown in Table 3. As expected, based on a priori stratification, very preterm neonates had greater cardiorespiratory instability as indicated by a number of these variables. As in our previous studies, a relationship between blood pressure and microvascular flow was observed in neonates <37 weeks gestational age (*P* = 0.006, *r* = −0.30). When split for sex, this relationship was observed in females only (*P* = 0.002, *r* = −0.50; males: *P* = 0.42, *r* = −0.12), suggesting some disconnect between central and peripheral cardiovascular regulation in male neonates.

As in our previous studies (Stark et al. ^32^), clinical illness severity as determined by the CRIB II score was correlated with microvascular blood flow at 24 h postnatal age (*P* = 0.003, *r* = 0.35). A correlation with 6 h microvascular blood flow was also observed (*P* = 0.02, *r* = 0.27). No relationship between CRIB score and microvascular blood flow was observed at 72 h postnatal age (*P* = 0.83, *r* = 0.03).

### Guinea pig neonates

Physical characteristics for guinea pig neonates are presented in Table 4. Gestation length in our guinea pig population is approximately 71 days (Palliser et al. ^24^). Spontaneous delivery in this cohort (*n* = 5) occurred at 70–71 days gestation. Caesarean section term delivery (*n* = 8) was carried out at 68–69 days gestation. Preterm delivery (*n* = 11) was performed at 61–63 days gestational age. There were no differences in gestational age at birth, birth weight, or monitoring score between sexes in any gestational age group. As expected, based on a priori stratification, preterm animals had significantly lower birth weight (males *P* = 0.02, females *P* = 0.03) and lower average neonatal monitoring scores indicating poorer physiological stability (*P* < 0.0001) than caesarean section delivered term controls. There was no difference in weight or average neonatal monitoring score over the study period between spontaneously delivered and caesarean section delivered term animals.

**Table 4.** Physical characteristics of guinea pig neonates.

	Preterm		Caesarean section term		Spontaneously delivered term	
	Female (*n*=8)	Male (*n*=8)	Female (*n*=8)	Male (*n*=8)	Female (*n*=4)	Male (*n*=4)
Gestational age (days)	62 (61–63)	62 (61–63)	68.5 (68–69)	69 (68–69)	71 (70–71)	70.5 (70–71)
Birth weight (g)	64.9 (43.7–79.4)‡	64.3 (57.2–88.0)‡	74.6 (67.2–103.3)	89.9 (58.8–106.3)	91.9 (74.0–102.8)	112.3 (88.4–126.6)
IUGR (*n*, %)	2 (25%)	0	2 (25%)	2 (25%)	1 (25%)	0
Neonatal monitoring score	8.6 (6.2–9.9)‡	8.2 (6.9–10.1)‡	11.5 (10.7–11.9)	11.4 (11.2–11.8)	11.7 (11.3–11.9)	11.8 (11.4–11.9)

Data are presented as median (range) or number (%) for pups surviving 24 h study period. Neonatal monitoring score presented is average score over study period. IUGR, intrauterine growth restriction.

‡ Significant difference between preterm and term caesarean section‐delivered pups, *P *< 0.05 within sex.

#### Guinea pig microvascular studies

##### Mode of delivery at term

Over the 24 h study period, microvascular blood flow was not different between spontaneously delivered and caesarean section delivered term animals (*P* = 0.63). There were no differences identified between sexes in the spontaneously delivered cohort (data not shown) or the caesarean section delivered cohort (outlined below). As mode of delivery demonstrated no effect at term, all further data referring to term and comparisons between gestational ages refer to caesarean section delivered term neonates only. Each group is thus represented by a minimum of 8 L, with only one dam represented by more than one male and one female pup (preterm animals: two females from same litter).

##### Effect of sex

**Figure 3.**
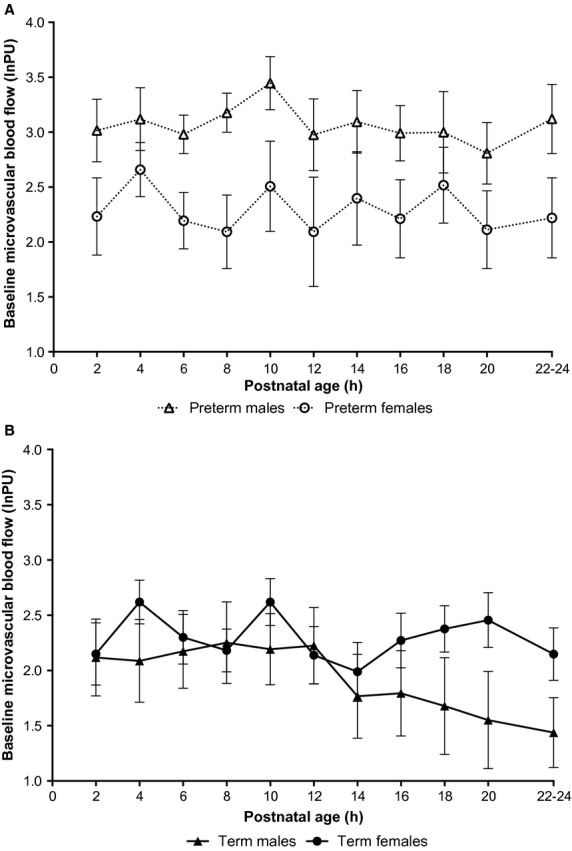
Microvascular blood flow over first day of life in neonatal guinea pigs. (A) Microvascular blood flow was significantly higher in male preterm neonates than in female preterm neonates over the study period (*P *= 0.01). (B) No differences between male and female term neonates were observed. Microvascular blood flow is expressed as log of arbitrary perfusion units (log_e_PU) and is presented as mean ± SEM.

In preterm animals, males had significantly higher microvascular blood flow than females over the study period (*P* = 0.01; Fig. 3a). No sex differences in microvascular blood flow were observed in term animals (*P* = 0.37; Fig. 3b).

##### Effect of gestational age

**Figure 4.**
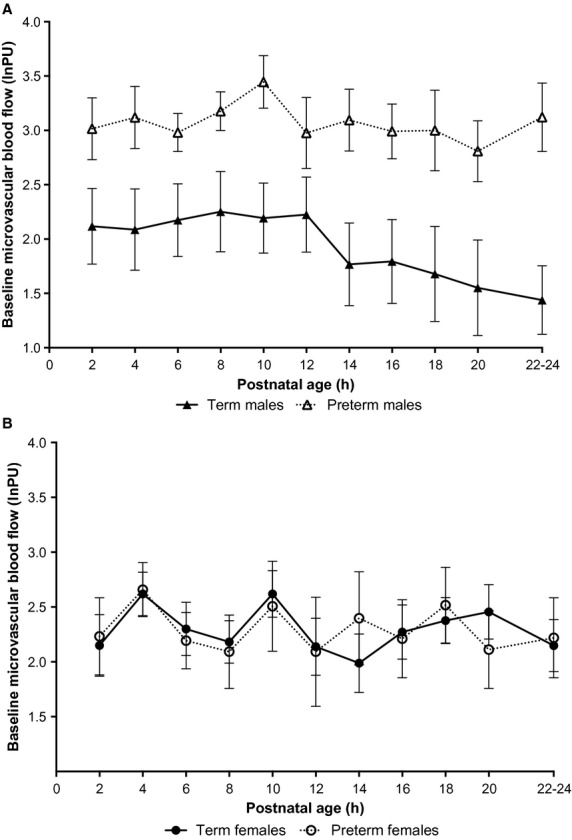
Microvascular blood flow is significantly higher in preterm than term newborn animals. (A) In male animals, preterm neonates had significantly higher microvascular blood flow than term animals over the 24 h study period (*P *= 0.003). (B) No gestational‐age‐dependent differences were observed in females. Microvascular blood flow is expressed as log of arbitrary perfusion units (log_e_PU) and is presented as mean ± SEM.

In male animals, significantly higher microvascular blood flow was observed in preterm animals compared to term controls (*P* = 0.003; Fig. 4a). This difference was greatest at 10 and 24 h postnatal age (*P* = 0.008 and *P* = 0.002, respectively). The difference at 10 h appears to be a result of an increase in blood flow in the preterm animals, with no change in term blood flows, whereas the difference at 24 h appears to be due to a decrease in term blood flows, with no change in preterm blood flows. Therefore, it appears that there is an interaction between gestational‐ and postnatal‐age in male animals: term blood flow decreased with advancing postnatal age, whereas in preterm males there was an initial increased flow which was maintained for at least 24 h postnatally (*P* = 0.03). No gestational‐age‐dependent differences in microvascular blood flow were observed in female animals (*P* = 0.98; Fig. 4b).

##### Effect of postnatal age

As outlined above, baseline microvascular blood flow increased after birth in male preterm animals, reaching a peak at 10 h postnatal age (*P* = 0.077). Blood flow then steadily decreased, returning to delivery levels by 24 h (*P* = 0.66). No such cycle was observed in female preterm animals or term male or female animals, however, it should be noted that there was a gradual decrease in blood flow after 12 h postnatal age observed in male term animals (*P* = 0.022).

## Discussion

These findings do not support a hypoperfusion–reperfusion cycle in the microvasculature over the first 24 h of extrauterine life in humans or guinea pigs. We observed high levels of baseline microvascular blood flow in preterm compared to term human neonates at both 6 and 24 h postnatal age. In both humans and guinea pigs, there was some evidence of an increase in flow following birth in the highest risk subgroup (preterm males), however, we do not believe this represents a hypoperfusion–reperfusion cycle as there was no cycle of vasoconstriction and vasodilation, as would be expected in this physiological response. Overall, blood flows were consistently higher in preterm males than term males, supporting this conclusion. Maximal flows were observed at 10 h postnatal age in the male preterm guinea pig and this is thought to represent the overall loss of peripheral vascular resistance seen in the preterm human at 24 h postnatal age. We do not know in humans whether the high blood flow seen at 24 h represents maximal microcirculatory flow or whether this occurs at an equivalent earlier time point as observed in the guinea pig. These findings are supported by the observations of others showing that peripheral blood flow increases over the first 24 h of postnatal age, without changes in systemic blood flow in small groups of preterm very low birth weight neonates (Ishiguro et al. ^15^).

The hypothesis of a hypoperfusion–reperfusion cycle led to speculation that adverse outcomes associated with cardiovascular compromise in the preterm neonates could be the result of ischemia–reperfusion injury induced by low systemic blood flow followed by decreased peripheral vascular resistance and thus high microvascular flow. However, our findings do not support the presence of such a cycle, and the mechanisms by which high blood flow may be involved in the development of adverse outcome are yet to be elucidated. One potential mechanism is through the loss of preload due to low peripheral resistance. It has now been shown that the preterm heart behaves similar to the adult failing heart: the preterm heart lacks the functional capacity to acutely adapt to changes in afterload and requires significant preload reserve in order to function effectively (Takahashi et al. ^36^; Osborn et al. ^23^; Eiby et al. ^8^). In line with this, we observed a basic relationship between LVO and microvascular blood flow at both 6 and 24 h postnatal age, which could be interpreted as either the pump pressure from the heart determining flow or that resistance in the peripheral microvasculature determines the output of the heart. The latter model is supported by the work of Eiby et al. (^8^).

Another potential mechanism is through inappropriate distribution of blood flow. The changes in microvascular blood flow reported in this study were measured in the skin (subcutaneous microcirculation). However, it is becoming increasingly clear that the evaluation of each organ's perfusion status at birth will be vital for understanding the mechanisms of inappropriate blood flow distribution and ultimately for the successful management of cardiovascular or circulatory compromise. Here, the subcutaneous microcirculation was investigated as representative of systemic microvascular flow. The subcutaneous circulation is, however, also a highly specialized system in its own right, so may vary significantly from other organ systems and tissues. Recently, others have also used the cutaneous microcirculation as an easily accessible and representative vascular bed for investigating the mechanisms underlying microcirculatory function and dysfunction. Several studies, including heart and renal disease studies, have utilized the skin as a model of circulation for investigating the vascular mechanisms underlying disease states, and suggest that the cutaneous circulation can be used as a marker of normal and impaired vascular control and adequacy of global blood flow (Wright et al. ^39^; Holowatz et al. ^14^; Stark et al. ^32^,b; Sahni et al. ^28^; Ishiguro et al. ^15^). Noninvasive techniques are required for clinical and routine use, and were employed in this study to allow for the direct comparison between guinea pigs and findings from clinical studies performed in preterm human neonates. Other technologies, such as Near‐Infrared Spectroscopy, which allow for detection of tissue‐oxygenation deficits and impaired microvascular reactivity as well as classification of disease severity, have been used successfully for the noninvasive characterization of microvascular behavior in microcirculation beds other than the skin (Nanas et al. ^22^). Having now shown the concordance of our human and animal studies, future regional microvascular studies can be undertaken in the guinea pig model.

Hemodynamically significant PDA was not associated with microvascular function in this study. Although PDA is a significant independent risk factor for circulatory compromise following preterm birth, previous studies have shown that sPDA is not associated with microvascular function in the immediate postnatal period (first 48 h) (Stark et al. ^32^). However, after this at 3–4 days postnatally, sPDA has a profound effect on the microcirculation in preterm infants: the persistent left‐to right shunting through the duct results in reduced blood volume in the periphery and thus systemic hypoperfusion and significantly lower functional vessel density (FVD) (Hiedl et al. ^13^). This results in major flow redistribution with a shift in perfusion away from larger vessels toward the microcirculation, further highlighting the contribution of the microvasculature to circulatory compromise and poor outcome in the preterm neonate.

Across species, preterm male neonates had consistently higher microvascular blood flow than term male neonates. In female human neonates, preterm microvascular blood flow was similar to term by 72 h postnatal life, whereas no differences between female term and preterm guinea pigs were seen throughout the 24 h study period. What gives rise to the sexual dimorphism in control of peripheral microvascular tone in the preterm newborn remains unknown, but may be associated with antenatal glucocorticoid exposure. Steroids are important mediators of the preterm human neonatal cardiovascular system, particularly the microvasculature, with effects on vascular tone, permeability, and endothelial integrity. It has been demonstrated that both long‐ and short‐term exposure to glucocorticoids alter vascular function (Clifton et al. ^6^) and betamethasone is also known to decrease placental vascular resistance (Wallace and Baker ^38^). In the newborn preterm female, antenatal glucocorticoid exposure is associated with greater autonomic activity and a state of overall peripheral vasoconstriction, and thus greater physiological stability in comparison to male newborns of the same gestational age (Stark et al. ^35^). Our group have demonstrated steroid involvement in a number of perinatal vascular pathways and this is in line with known effects of steroid dose and timing on smooth muscle cGMP production (van Bel et al. ^4^), carbon monoxide production (Choi and Alam ^5^; Stark et al. ^34^), and catecholamine and endothelin (ET)‐1 levels (Sabban and Kvetnansky ^27^). Elevated plasma levels of cGMP have been positively correlated with hypotension and severity of IVH in the premature neonate (van Bel et al. ^4^). Others have shown that prenatal glucocorticoids increase ET‐1 sensitivity and ETa receptor expression and binding, resulting in a greater degree of vasoconstriction in response to ET‐1 (Trevisi et al. ^37^). Current clinical practice is administration of antenatal corticosteroids to women at risk of preterm delivery. As such, the majority of preterm neonates in the human study (70%) and all neonates in the animal study were exposed to a full course of antenatal glucocorticoids. It is therefore not clear whether microvascular dysregulation in the initial extrauterine period is linked to betamethasone exposure. Future animal experiments, where betamethasone can be withheld, may help to answer such questions.

Unlike in previous studies, we did not observe a significant low systemic flow state in the first hours of postnatal life. Earlier reports observed low SVC flow in one‐third of neonates delivered prior to 30 weeks completed gestation (Kluckow and Evans ^18^). More recent studies have demonstrated much lower rates (13% in one recent study from Australia, Lakkundi et al. ^20^), comparable with incidence seen in this study. The lower incidences of low SVC in the more recent studies may relate to different ventilation strategies: mechanical ventilation appears to be associated with low SVC flow (Evans and Kluckow ^11^; Kluckow and Evans ^18^; Miletin and Dempsey ^21^). A discussion relating to the relationship between mechanical ventilation and transitional hemodynamics has been recently published (Lakkundi et al. ^20^).

This comparative study has demonstrated that human and animal studies of cardiovascular transition are consistent: there is a period of high microvascular flow after birth, however, no period of low flow (hypoperfusion stage) precedes this, suggesting that no hypoperfusion–reperfusion cycle exists during early extrauterine life. The data strongly support the use of the preterm guinea pig in future studies to investigate the mechanisms underlying regulation of microvascular function following birth. To better characterize the control of microvascular blood flow in early extrauterine life and to understand how this may contribute to cardiovascular compromise and adverse outcome, investigation of physiologically relevant vasoactive molecules and characterization of the microvessels during this time is required. Videomicroscopy may help to examine microvascular structure (Kroth et al. ^19^) and intervention studies in animals will allow further investigation of the interrelationship between cardiac output, preload, afterload, microvascular flow, and SVC flow. Elucidation of these processes may then aid clinicians in managing the circulatory dysfunction of preterm neonates, particularly those at greatest risk, preterm male neonates.

## Conflict of Interest

None declared.
